# Modeling and analyzing V2V communication limitations impacts on connected and automated vehicle platoons

**DOI:** 10.1371/journal.pone.0328555

**Published:** 2025-08-18

**Authors:** Yulu Dai, Liang Hu, Yanbin Liu, Aixi Yang

**Affiliations:** 1 Hangzhou Vocational and Technical College, Hangzhou, Zhejiang, China; 2 Anhui Province Key Laboratory of Intelligent Car Wire-Controlled Chassis System, Wuhu, China; 3 College of Civil and Transportation Engineering, Hohai University, Nanjing, Jiangsu, China; 4 Tsinghua University, Beijing, China; 5 Zhejiang University, Hangzhou, Zhejiang, China; Beijing Institute of Technology, CHINA

## Abstract

Connected and automated vehicle (CAV) platooning, enabled by Vehicle-to-Vehicle (V2V) communication, promises significant improvements in traffic safety, throughput, and energy efficiency. However, communication constraints — such as range limitations and intermittent connectivity — disrupt information flow, destabilizing platoon dynamics. Existing models lack a unified framework to analyze how these constraints propagate through CAV interactions. To address this gap, the Platoon Intelligent Driver Model (PIDM) is proposed, a novel analytical framework that integrates dynamic communication topologies (predecessor-following, predecessor-leader-following, and 𝕜-predecessor-leader-following) with vehicle dynamics under V2V limitations. The PIDM enables systematic stability analysis and quantifies disturbance propagation mechanisms. Through numerical simulations, the study demonstrates that: (1) 𝕜-predecessor-leader-following topology reduces recovery time by 32% compared to conventional topologies; (2) smaller communication ranges (4–6 vehicles) optimize stability in urban roads, while larger ranges (8–10 vehicles) suit highways; (3) delay time tolerance thresholds depend on platoon size and topology complexity. These findings provide actionable guidelines for designing robust CAV platoon systems under real-world communication constraints.

## 1. Introduction

Connected and automated vehicle (CAV) platooning promises transformative improvements in traffic efficiency, safety, and energy sustainability by leveraging real-time coordination through Vehicle-to-Vehicle (V2V) communication [1,2]. However, practical deployment faces a critical yet understudied challenge: communication constraints—such as limited range, intermittent connectivity, and latency—destabilize platoon dynamics by disrupting information flow [3,4]. While existing studies optimize platoon control under idealized communication (e.g., [5–7]), they largely ignore how dynamic communication topologies adapt to real-world V2V limitations. This gap undermines the reliability of platoon systems in scenarios where communication range restricts vehicle interactions or delays amplify disturbances.

For instance, optical V2V-based platooning [8] enhances stability but assumes uninterrupted communication, while energy-saving strategies for multi-lane platoons [9] overlook topology fragmentation caused by range constraints. Similarly, classical models like the Intelligent Driver Model (IDM) [10] focus on single-predecessor interactions, failing to capture multi-vehicle coordination under distance-limited V2V. These oversights are problematic: fragmented platoons due to bandwidth limitations or delayed responses from distant predecessors can trigger cascading speed oscillations, jeopardizing safety and efficiency [11,12]. A unified framework to model and analyze these effects is urgently needed.

The merits of applying V2V communication in promoting CAV platoons’ safety have been explored in the literature [7–11]. On the other hand, V2V communication limitations could constrain the configuration of platoon size (i.e., the maximum allowable platoon size) which has an essential impact on traffic [12–16]. The maximum number of vehicles in a platoon is often restricted by bandwidth limitations, leading to fragmented multi-platoon formations that degrade traffic efficiency. For instance, [15] proposed a multi-lane energy-saving strategy for connected electric vehicles but did not address how dynamic communication topologies adapt under such constraints. Simulation results and field experiments reveal that a platoon with a larger size is of assistance better reap the benefits provided by platooning technology [17–19], while it is burdensome for communication system when the length of the platoon is large [20]. According to previous studies [14,21,22], V2V communication limitations can be divided into two types: communication range limitation and communication distance limitation. The former limits the number of vehicles in a platoon for management; and the latter constrains other CAVs information transferring distance due to the capacity of V2V communication technology. In addition, a CAV platoon is formed according to the applied information flow topology which is closely related to the way a vehicle obtains the information of its surrounding vehicles. As a consequence of relative research [2,4,18,23–25], types of information flow topologies generated by V2V communications can be summarized into three categories: predecessors following type, two-predecessor-leader following type, and 𝕜-predecessors following type, from the viewpoint of information receiver. To date, most current theoretical studies on CAV platoon management have investigated the influence of the limitation of communication on platoon string stability [26,27]. Nevertheless, to the best of our knowledge, there exists little literature which connects CAVs’ dynamics with the potential cooperative performances and considers platoon communication based on information flow topology that can be affected by V2V communication limitations.

While V2V communication significantly enhances CAV platoon safety through real-time coordination [21,28,29], its inherent limitations impose critical constraints on system scalability and operational efficiency. Current research reveals a fundamental trade-off: although larger platoons improve traffic throughput and energy efficiency [30], they exacerbate communication burdens through increased packet loss and time delays [31,32]. Time delays in V2V data transmission can amplify disturbances in platoons, as shown by [28], who developed an interacting multiple model-based ETUKF for state estimation but assumed ideal communication conditions. Despite these advancements, existing models rarely integrate dynamic communication topologies with vehicle dynamics, leaving a gap in understanding how V2V limitations propagate through CAV platoons. Therefore, modeling the driving behavior of CAV platoons within V2V communication limitations in the simulation environment is a challenging task.

To address this, we propose the Platoon Intelligent Driver Model (PIDM), the first analytical framework integrating dynamic communication topologies (predecessor-following (PF), predecessor-leader-following (PLF), and 𝕜-predecessor-leader-following (𝕜-PLF)) with vehicle dynamics under V2V constraints. Our key contributions are:

1) PIDM explicitly models how communication range and distance limitations reshape information flow, enabling analysis of disturbance propagation.
2) We derive rigorous stability criteria linking communication parameters (e.g., range S, delay τ) to platoon resilience, filling a theoretical gap in existing literature.
3) Through simulations across highway, urban and heterogeneous traffic flow scenarios, we demonstrate that smaller platoons (4–6 vehicles) optimize urban stability, while larger ranges (8–10 vehicles) suit highways—a finding critical for adaptive V2V design.

The remainder of the paper is organized as follows. A brief background and problem description are given in the next section. Section 3 presents three typical information flow topologies and proposes the PIDM to describe the manner CAVs under V2V communication limitations. Section 4 conducts a series of numerical experiments that demonstrate how CAVs are influenced by communication limitations via three typical kinds of information flow topologies. Section 5 discusses the limitation of this study. In the end, Section 6 concludes this paper and suggests future research directions.

## 2. V2V communication topologies for CAV platoons

**Fig 1:**
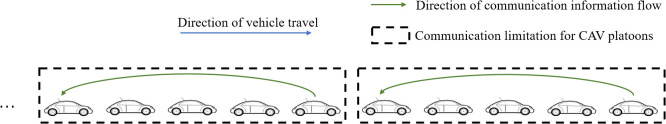
Schematic of CAV platoons with communication limitations.

In general, CAVs exchange information by means of radars transmission or wireless broadcast [28]. Nevertheless, the formation of a CAV platoon mainly depends on the V2V protocol which has communication limitations. For the sake of avoiding forced communication abnormalities, CAVs tend to form multi-platoon when the number of them goes beyond the limit of the maximum V2V communication bandwidth as depicted in Fig 1. In this section, first, the assumptions for CAV platoons is presented. Then, in this scenario, three typical dynamic information flow topologies considering V2V communication limitations are elaborated on.

### 2.1. Assumptions

This study aims to study the impact of V2V communication limitations on the safety and efficiency of a CAV platoon. V2V communication limitations are referred to as communication range limitation and communication distance limitation, specifically. The assumptions of this study are put forward as follows:

Assumption 1: All vehicles in the platoon are identical connected and automated vehicles (CAVs) under 100% market penetration, eliminating human-driven vehicle (HDV) variability and ensuring uniform dynamic characteristics.

Assumption 2: Platoons operate on a flat, single-lane road segment without lane-changing, overtaking, or cut-in/out maneuvers, focusing solely on longitudinal dynamics under uninterrupted flow conditions.

Assumption 3: The PF topology serves as the foundational communication scheme, where each CAV receives information only from its immediate predecessor [33–35]. Extended topologies (PLF, 𝕜-PLF) are analyzed as generalized cases to evaluate multi-vehicle information integration.

Assumption 4: The study isolates V2V communication range/distance limitations as the primary constraint, excluding packet loss, signal noise, cybersecurity threats, or other channel imperfections. Information transmission is assumed error-free but restricted by predefined range (N vehicles) or distance (S meters).

Assumption 5: A uniform communication delay (τ = 0.2s) is applied to all CAVs, consistent with the simulation parameters in Table 2, to decouple delay effects from topology-dependent dynamics.

### 2.2. Information flow topologies for CAVs

**Fig 2:**
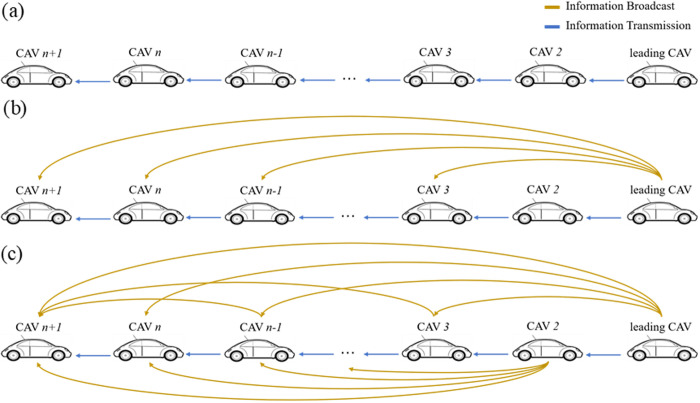
Schematic diagram of information flow topologies for CAVs. (a) Predecessor following topology, (b) Predecessor-leader following topology, (c) 𝕜 predecessor-leader following topology.

There are n CAVs in one platoon including a leader (noted as the leading CAV) and n-1 followers (noted as CAV 2 – CAV n), based on the V2V communication limitations, and based on ad-hoc network performs dynamic information flow topology settings for the leader of the platoon for the sake of not losing communication completely, as shown in Fig 2. The platoon can have different information flow topologies, either broadcast-based or transmission-based. Actually, V2V communication might generate various types of information flow topologies for CAVs. As CAVs move forward, each CAV is generally only concerned with vehicles’ manners ahead but not behind [30,31]. Fig 2 shows three typical information flow topologies that are employed in this study, many other topologies are not considered here, but they all can be analyzed leveraging similar approaches. Note that, the information flow topology between platoons is Predecessor following topology.

The three types of information flow topologies are as follows:

(a) Predecessor following topology — PF topology;
(b) Predecessor-leader following topology— PLF topology
(c) 𝕜 predecessor-leader following topology — 𝕜-PLF topology.

More specifically, PF topology indicates that information of each CAV is only intended to be delivered to the vehicle immediately following it; PLF topology is that the followers receive information from its immediate predecessor and the leader in the platoon; meanwhile, 𝕜-PLF topology is that the followers receive information from all vehicles ahead in the platoon. Furthermore, PLF and 𝕜-PLF topologies enable multi-vehicle cooperative driving, whereas PF topology is adaptable for CAV decentralized control. To better understand V2V communication limitations impacts on CAVs, a graph is utilized to model the information flow topology among CAVs. In detail, the information flow topology is defined as a directed graph = ( Φ, Ψ, Θ ) with node set Φ, edge set Ψ, and weight set Θ. Each node in set Φ={1, 2,3⋯N} stands for a CAV in the platoon, whereas the edge set Ψ⊆Φ×Φ stands for the information exchange between a pair of CAVs. Note that, an edge (i,j) is existent if CAV i obtains information from CAV j; otherwise, the edge will not be existent. Apart from that, one vehicle cannot obtain its own messages through V2V [26,30], as well as the CAVs behind. To facilitate the analysis, a binary variable $\varpi_{i,j}=\{0,1\}$ is used to define whether directional communication connection (i,j) is existent. Therein to, $\varpi_{i,j}=1$ corresponds to establishing communication connection, and $\varpi_{i,j}=0$ corresponds to no communication connection. According to this notation, we can construct a lower triangular adjacency matrix owing to the assumption that the information is from the vehicles ahead. The adjacency matrix is elaborated as:


$$W(t)=\begin{array}{c} \begin{array}{cc} & \;\;\;\;\;\;\begin{array}{cc} 1 & \begin{array}{cc} \;2 & \begin{array}{cc} \cdots & \begin{array}{cc} \;j & \;\begin{array}{cc} \cdots & \begin{array}{cc} \;N-1 & N \end{array} \end{array} \end{array} \end{array} \end{array} \end{array} \end{array}\\ \begin{array}{c} \begin{array}{c} \begin{array}{c} \begin{array}{c} \begin{array}{c} 1\\ 2\\ 3 \end{array}\\ \vdots \end{array}\\ i \end{array}\\ \vdots \end{array}\\ N \end{array}[\begin{array}{cc} \begin{array}{ccc} 0 & 0 & \cdots \\ \varpi_{21} & 0 & \cdots \\ \varpi_{31} & \varpi_{32} & 0 \end{array} & \begin{array}{ccc} 0\; & \cdots & \begin{array}{cc} \;0\; & 0 \end{array}\\ 0 & \cdots & \;\begin{array}{cc} 0 & \;0 \end{array}\\ \ddots & \cdots & \;\begin{array}{cc} \vdots & \;0 \end{array} \end{array}\\ \begin{array}{ccc} \vdots & \vdots & \ddots \\ \varpi_{i1} & \varpi_{i2} & \cdots \\ \begin{array}{c} \vdots \\ \varpi_{N1} \end{array} & \begin{array}{c} \vdots \\ \varpi_{N2} \end{array} & \begin{array}{c} \vdots \\ \cdots \end{array} \end{array} & \begin{array}{ccc} \ddots & \ddots & \;\begin{array}{cc} \vdots & \;\vdots \end{array}\\ \varpi_{ij} & 0 & \begin{array}{cc} 0 & \;0 \end{array}\\ \begin{array}{c} \vdots \\ \varpi_{Nj} \end{array} & \begin{array}{c} \ddots \\ \cdots \end{array} & \begin{array}{cc} \begin{array}{c} \ddots \\ \varpi_{N,N-1} \end{array} & \begin{array}{c} \vdots \\ 0 \end{array} \end{array} \end{array} \end{array}{]}_{N\times N} \end{array}$$ (1)

The adjacency matrix W(t) represents the communication topology within a platoon. Each element $\varpi_{i,j}$ is a binary variable indicating whether CAV *i* receives information from CAV *j*:


$$\varpi_{i,j}=\left\{ \;\;\begin{array}{c} 1\;if\;CAV\;i\;communicates\;with\;CAV\;j\;at\;time\;t\\ 0\;otherwise \end{array}\right.\;$$ (2)

For a platoon with *N* CAVs, W(t) is structured as a lower triangular matrix, since communication is unidirectional (only preceding vehicles transmit information). More specifically, the expression of PLF and 𝕜-PLF topologies are given by Eqs. (3) and (4), respectively.


$$W_{PLF}(t)=\begin{array}{c} \begin{array}{cc} \; & \begin{array}{cc} \;\;\;\;\;\;\;\;\;\;1 & \begin{array}{cc} 2 & \begin{array}{cc} \cdots & \begin{array}{cc} j & \;\begin{array}{cc} \cdots & \begin{array}{cc} \;N-1 & \;N \end{array} \end{array} \end{array} \end{array} \end{array} \end{array} \end{array}\\ \begin{array}{c} \begin{array}{c} \begin{array}{c} \begin{array}{c} \begin{array}{c} 1\\ 2\\ 3 \end{array}\\ \vdots \end{array}\\ i \end{array}\\ \vdots \end{array}\\ N \end{array}[\begin{array}{cc} \begin{array}{ccc} 0 & 0 & \cdots \\ 1 & 0 & \cdots \\ 1 & 1 & 0 \end{array} & \begin{array}{ccc} 0\; & \cdots & \;\begin{array}{cc} 0 & \;0 \end{array}\\ 0\; & \cdots & \;\begin{array}{cc} 0 & \;0 \end{array}\\ \ddots & \cdots & \;\begin{array}{cc} \vdots & \;0 \end{array} \end{array}\\ \begin{array}{ccc} \vdots & \vdots & \ddots \\ 1 & 0 & \cdots \\ \begin{array}{c} \vdots \\ 1 \end{array} & \begin{array}{c} \vdots \\ 0 \end{array} & \begin{array}{c} \vdots \\ \cdots \end{array} \end{array} & \begin{array}{ccc} \ddots & \ddots & \;\begin{array}{cc} \vdots & \;\vdots \end{array}\\ 1 & 0 & \;\begin{array}{cc} 0 & \;0 \end{array}\\ \begin{array}{c} \vdots \\ 0 \end{array} & \begin{array}{c} \ddots \\ \cdots \end{array} & \;\begin{array}{cc} \begin{array}{c} \ddots \\ 1 \end{array} & \;\begin{array}{c} \vdots \\ 0 \end{array} \end{array} \end{array} \end{array}{]}_{N\times N} \end{array}$$ (3)

$$W_{k-PLF}(t)=\begin{array}{c} \begin{array}{cc} \; & \begin{array}{cc} \;\;\;\;\;\;1 & \begin{array}{cc} 2 & \begin{array}{cc} \cdots & \begin{array}{cc} j & \;\begin{array}{cc} \cdots & \begin{array}{cc} \;N-1 & \;N \end{array} \end{array} \end{array} \end{array} \end{array} \end{array} \end{array}\\ \begin{array}{c} \begin{array}{c} \begin{array}{c} \begin{array}{c} \begin{array}{c} 1\\ 2\\ 3 \end{array}\\ \vdots \end{array}\\ i \end{array}\\ \vdots \end{array}\\ N \end{array}[\begin{array}{cc} \begin{array}{ccc} 0 & 0 & \cdots \\ 1 & 0 & \cdots \\ 1 & 1 & 0 \end{array} & \begin{array}{ccc} 0\; & \cdots & \;\begin{array}{cc} 0 & \;0 \end{array}\\ 0\; & \cdots & \;\begin{array}{cc} 0 & \;0 \end{array}\\ \ddots & \cdots & \;\begin{array}{cc} \vdots & \;0 \end{array} \end{array}\\ \begin{array}{ccc} \vdots & \vdots & \ddots \\ 1 & 1 & \cdots \\ \begin{array}{c} \vdots \\ 1 \end{array} & \begin{array}{c} \vdots \\ 1 \end{array} & \begin{array}{c} \vdots \\ \cdots \end{array} \end{array} & \begin{array}{ccc} \ddots & \ddots & \;\begin{array}{cc} \vdots & \;\vdots \end{array}\\ 1 & 0 & \;\begin{array}{cc} 0 & \;0 \end{array}\\ \begin{array}{c} \vdots \\ 1 \end{array} & \begin{array}{c} \ddots \\ \cdots \end{array} & \;\begin{array}{cc} \begin{array}{c} \ddots \\ 1 \end{array} & \;\begin{array}{c} \vdots \\ 0 \end{array} \end{array} \end{array} \end{array}{]}_{N\times N} \end{array}$$ (4)

For the sake of simplicity, no noise, such as sensor errors or communication delay time, is considered as we aim to first highlight the potential influence brought by V2V communication limitations. In a cooperative platoon with information flow topology, every single CAV should adjust its speed/ acceleration at the same time step based on other CAVs. In order to investigate the effects of V2V communication limitations, modeling the dynamics of CAVs in a platoon is the key of our study which is developed in Section 3.

## 3. Modeling CAV dynamics under V2V communication limitations

In this section, we model CAV dynamics under V2V communication limitations through modification of a CAV car-following model that is considered as a microscopic cyber-physical system comprised of communication and vehicle subsystems. Table 1 lists the variables and parameters utilized hereafter.

**Table 1:** Variables and parameters description.

Category	Variables and parameters	Definition
Kinematic Parameters	$a_{i}(t)$	Acceleration of the subject CAV i at time t (m/s^2^)
	$v_{i}(t)$	Speed of the subject CAV i at time t (m/s)
	α	Maximum desired acceleration (m/s^2^)
	β	Maximum desired deceleration (m/s^2^)
Communication Constraints	*N*	Maximum number of CAVs in a platoon (range limitation)
	*S*	Maximum communication radius (distance limitation) (m)
Dynamic Weights	$\sigma_{i}$	Weight for space headway from CAV *i*
	$\delta_{i}$	Weight for speed difference from CAV *i*

**Table 2:** Simulation parameters.

Scenario	Symbol	Value	Scenario	Symbol	Value
Freeway	α	1m/s^2^	City Road	α	1m/s^2^
	$s_{0}$	2m		$s_{0}$	2m
	β	2m/s^2^		β	2m/s^2^
	δ	4		δ	4
	$v_{f}$	120 km/h		$v_{f}$	72 km/h
	*T*	0.5s		*T*	1.2s
	τ	0.2s		τ	0.2s
	$\lambda_{1}$	0.1		$\lambda_{1}$	0.1
	$\lambda_{2}$	1		$\lambda_{2}$	1
	ρ	0.01		ρ	0.01

### 3.1. CAV dynamics under V2V communication limitation

To describe V2V communication limitation effects on CAV platoons, microscopic vehicular dynamics that describe individual CAV car-following behaviors via gaps, velocity, speed difference, and acceleration are concentrated on in this study.

In general, the purpose of CAV platoon is that the following vehicles could still maintain the same speed (i.e., equilibrium speed) with the leader and hold a desired organization depended on inter-vehicle spacing policy under small perturbations. This CAV platoon objective can be formed as Eq. (5):


$$\left\{ \begin{array}{c} \;\;\;\;\;\;\;\;\;\;\;\;\;\;\;\;\lim_{t\to \infty }\left\|v_{i}(t)-v_{0}(t)\right\|=0\\ \lim_{t\to \infty }\left\|x_{i}(t)-x_{j}(t)-\Delta s_{i,j}^{*}\right\|=0 \end{array}\right.\;$$ (5)

where $\Delta s_{i,j}^{*}$ is the desired space between CAV *i* and CAV *j* determined by the formation geometry of CAV platoons and is usually set as equilibrium distance gap. Moreover, the acceleration function $a_{i}(t)$ in terms of the gap ${\Delta s}_{i}(t)$ and the speed difference $\Delta v_{i}(t)$ to the leading vehicle, and the vehicle’s speed $v_{i}(t)$ can stand for the vehicle’s response in a time-continuous car-following model, and formulated as a set of ordinary differential equations (ODEs):


$$a_{i}(t)=\frac{\text{d}v_{i}(t)}{\text{d}t}=f_{i}\left(v_{i}(t),{\Delta s}_{i}(t){,\Delta v}_{i}(t)\right)$$ (6)

where $f_{i}(\cdot )$ is a nonlinear function which can be specifically defined for different car-following models. After extending Eq. (6) with reaction time delay τ, the acceleration is of the general form:


$$a_{i}(t-\tau )=\frac{dv_{i}(t-\tau )}{dt}=f_{i}\left(v_{i}(t-\tau ),{\Delta s}_{i}(t-\tau ){,\Delta v}_{i}(t-\tau )\right)$$ (7)

Eq. (7) demonstrates that each vehicle’s acceleration is determined by own speed, clearance gap, and velocity difference with its predecessor. Denote *S* as the maximum communication radius under V2V communication distance limitation, and denote *N* as the maximum number of CAVs in a platoon under V2V communication range limitation. Under V2V communication distance limitation, the number of CAVs cooperating with CAV *i* is labeled by $M_{i}$ as:


$$M_{i}=\{j\epsilon \omega \backslash \left\{i\right\}:0<x_{j}-x_{i}\leq S\}$$ (8)

where ω is the set of platoon CAVs. Under V2V communication range limitation, the number of CAVs cooperating with CAV *i* is labeled by $H_{i}$ as:


$$H_{i}=\{\left\{i\right\}:0<i\leq N\}$$ (9)

In the V2V environment with PLF topology or 𝕜-PLF topology, the subject CAV can obtain information from multiple CAVs ahead, thus must not be ignored. According to relative research [31], by incorporating the communication limitation and reaction delay as a generalized implicit function, a CAV dynamics model is proposed as Eq. (10).


$$a_{i}(t)=f_{i}\left(v_{i}(t-\tau ),\sum_{j\in P_{i}}m_{j}\varpi_{i,j}{\Delta s}_{ij}(t-\tau ),{\sum_{j\in P_{i}}m_{j}\varpi_{i,j}\Delta v}_{ij}(t-\tau )\right)$$ (10)

$$\begin{array}{c} \sum {\mathrm{\backslash nolimits}}_{j\in P_{i}}m_{j}=1,\;{\Delta s}_{ij}=x_{i}-x_{j},\;{\Delta v}_{ij}=v_{i}-v_{j},\;P_{i}\in {[M}_{i},\;H_{i}],\;m_{j}\in [\sigma_{j},\delta_{j}] \end{array}$$

where $\sigma_{j}$ and $\delta_{j}$ represent weighting coefficients associated with the space headway and speed difference, respectively, which influence the subject CAV’s dynamics. The highlight of our proposed model is the introduction of dynamic communication topology structure, which varies due to the type of information flow topology. To account for that the communication range and its limitations for CAV platoons are directly affected by the communication scheme, we integrate the communication scheme into the modeling. Moreover, a logistic function is preferred as the mathematical formulation of weighting coefficient, (see the vast literature on discreet choice modeling, e.g., Hensher et al. [33], Train [34], A. Sharma [15], etc.). Based on this, both $\sigma_{j}$ and $\delta_{j}$ are shown in Eqs. (11) and (12):


$$\sigma_{j}=\frac{1}{1+e^{\lambda_{1}(x_{j}-S)}}/\sum_{j\in M_{i}}\frac{1}{1+e^{\lambda_{1}(x_{j}-S)}}$$ (11)

$$\delta_{j}=\frac{1}{1+e^{\lambda_{2}(j-1-N)}}/\sum_{j\in H_{i}}\frac{1}{1+e^{\lambda_{2}(j-1-N)}}$$ (12)

where $\lambda_{1}$, $\lambda_{2}$ are the parameters that govern the shape of the function. In particular, Eq. (10) is degraded into Eq. (7) when $M_{i}$ = 1 and $H_{i}$ = 1 with PF topology.

### 3.2. Vehicle dynamics specification using the Intelligent Driver Model (IDM)

The CAV dynamics are specified by using a vehicle dynamics model, with the goal of enabling detailed theoretical and numerical analyses. Literally, hundreds of car-following models have been developed over the decades. Among them, the Intelligent Driver Model (IDM) [35] has been widely used in controlling the longitudinal movements of CAVs for a CAV platoon system design. Additionally, field experiments have verified that CAVs that use the IDM provides more comfortable car-following behavior than other controllers [36]. In this study, the IDM is selected to simulate CAVs for it shows clearly intuitive physical meaning and provides collision-free behaviors and smooth traffic flow [1,37]. Furthermore, compared to other microscopic models, IDM is of great realism when capturing the dynamics of different congestion levels [30]. Furthermore, it is a sophisticated model that has been used to model the longitudinal dynamics of CAVs in many existing studies [22,38,39].

The formula of IDM with reaction delay can be described by the following equation:


$$a_{i}(t)=\alpha \left(1-{\left(\frac{v_{i}(t-\tau )}{v_{f}}\right)}^{\delta }-{\left(\frac{s\left(v_{i}(t-\tau ),{\Delta v}_{i}(t-\tau )\right)}{{\Delta s}_{i}(t-\tau )}\right)}^{2}\right)$$ (13)

and


$$s\left(v_{i}(t-\tau ),{\Delta v}_{i}(t-\tau )\right)=s_{0}+Tv_{i}(t-\tau )+\frac{v_{i}(t-\tau ){\Delta v}_{i}(t)}{2\sqrt{\alpha \beta }}$$

where T denotes the safety time headway.

The traditional IDM only considers the impacts of the immediate preceding vehicle as PF topology. However, it is insufficient to capture the CAV performance when each CAV exchanges information with multiple surrounding vehicles. Hence, a revised IDM incorporating cooperation through V2V communications, labeled platoon IDM (PIDM), is formulated as follow:


$$a_{i}(t)=\alpha \left(1-{\left(\frac{v_{i}(t-\tau )}{v_{f}}\right)}^{\delta }-{\left(\frac{s(.)}{{\Delta s}_{i}(t-\tau )}\right)}^{2}\right)+\rho \sum_{j\in P_{i}}k_{j}{\varpi_{i,j}(\Delta s}_{i,j}(t-\tau )-\Delta s_{i,j}^{*})$$ (14)

and


$$s(.)=s_{0}+Tv_{i}(t-\tau )+\frac{v_{i}(t-\tau ){\sum_{j\in P_{i}}k_{j}\varpi_{i,j}\Delta v}_{i,j}(t)}{2\sqrt{\alpha \beta }}$$

$$\Delta s_{i,j}^{*}=\sum_{m=j+1}^{i}{(s}_{0}+Tv_{m}(t-\tau )){\left(1-{\left(\frac{v_{m}(t-\tau )}{v_{f}}\right)}^{\delta }\right)}^{-1/2}$$

where k is σ or δ and $P_{i}$ is $M_{i}$ or $H_{i}$ according to the type of limitation. The latter part of Eq. (14) which is linear is used to revise the acceleration based on distance gap between other vehicles.

To investigating the adverse effects of V2V communication limitations, it is of significance to evaluate the stability of CAV platoon dynamics affected by small perturbations under V2V communication limitations over space. By definition, stability analysis is the ability of vehicles to react to small perturbations when the platoon is traveling within an equilibrium situation. In the equilibrium situation, the state of the subject vehicle can be calculated by $f_{i}\left(v_{i}^{e},g_{i}^{e},0\right)=0$. To rigorously validate the proposed PIDM, we analyze both asymptotic stability (local convergence to equilibrium). Linearizing the PIDM dynamics-around equilibrium states $v_{i}(t)=v_{i}^{e}{,\Delta s}_{i}(t)=g_{i}^{e}$, define the augmented state −1vector:


$$z_{i}(t)=[\begin{array}{c} \delta v_{i}(t)\\ {\delta s}_{i}(t) \end{array}],\;\delta v_{i}(t)=v_{i}-v_{i}^{e},\;\delta s_{i}(t)={\Delta s}_{i}-g_{i}^{e}$$

The system dynamics take the form:


$${\dot{z}}_{i}(t)=Az_{i}(t)+Bz_{i}(t-\tau )+Cz_{i-1}(t)+Dz_{i-1}(t-\tau )$$

$$A=[\begin{array}{cc} 0 & 0\\ -1 & 0 \end{array}],\;B=[\begin{array}{cc} f_{i}^{v}+f_{i}^{\Delta v}\sum_{j\in P_{i}}m_{j}\varpi_{i,j} & f_{i}^{g}\sum_{j\in P_{i}}m_{j}\varpi_{i,j}\\ 0 & 0 \end{array}],$$ (15)

$$C=[\begin{array}{cc} 0 & 0\\ 1 & 0 \end{array}],\;D=[\begin{array}{cc} -f_{i}^{\Delta v}\sum_{j\in P_{i}}m_{j}\varpi_{i,j} & -f_{i}^{g}\sum_{j\in P_{i}}m_{j}\varpi_{i,j}\\ 0 & 0 \end{array}],$$

where $f_{i}^{v},$ $f_{i}^{\Delta v}$and $f_{i}^{g}$ are partial derivatives from Eq. (A13) (the details can be seen in *Supporting information*), a Lyapunov functional $V(t)={z_{i}}^{T}Pz_{i}+\int_{t-\tau }^{t}{z_{i}}^{T}(s)Qz_{i}(s)ds+\tau \int_{-\tau }^{0}\int_{t+\theta }^{t}{{\dot{z}}_{i}}^{T}(s)R{\dot{z}}_{i}(s)dsd\theta$ ($P=P^{T}>0,\;Q=Q^{T}>0,\;R=R^{T}>0$), yields:


$$\dot{V}(t)=2{z_{i}}^{T}(t)P{{\dot{z}}_{i}}^{T}(t)+{z_{i}}^{T}(t)Qz_{i}(t)-{z_{i}}^{T}(t-\tau )Qz_{i}(t-\tau )+\tau^{2}{{\dot{z}}_{i}}^{T}(t)R{\dot{z}}_{i}(t)-\tau \int_{t-\tau }^{t}{{\dot{z}}_{i}}^{T}(s)R{\dot{z}}_{i}(s)ds$$ (16)

Approximate the integral term using Jensen’s inequality:


$$\tau \int_{t-\tau }^{t}{{\dot{z}}_{i}}^{T}(s)R{\dot{z}}_{i}(s)ds\geq {\left(\int_{t-\tau }^{t}{\dot{z}}_{i}(s)ds\right)}^{T}R\left(\int_{t-\tau }^{t}{\dot{z}}_{i}(s)ds\right)={\left(z_{i}(t)-z_{i}(t-\tau )\right)}^{T}R\left(z_{i}(t)-z_{i}(t-\tau )\right)$$ (17)

Hence,


$$\dot{V}\leq {\left[\begin{array}{c} \begin{array}{c} z_{i}(t)\\ z_{i}(t-\tau ) \end{array}\\ \begin{array}{c} z_{i-1}(t)\\ z_{i-1}(t-\tau ) \end{array} \end{array}\right]}^{T}Y\left[\begin{array}{c} \begin{array}{c} z_{i}(t)\\ z_{i}(t-\tau ) \end{array}\\ \begin{array}{c} z_{i-1}(t)\\ z_{i-1}(t-\tau ) \end{array} \end{array}\right]$$

$$\text{Y}=[\begin{array}{ccc} \begin{array}{cc} 2PA+\tau^{2}A^{T}RA-R & PB+\tau^{2}A^{T}RB+R\\ 0 & \tau^{2}B^{T}RB-R \end{array} & \begin{array}{c} PC+\tau^{2}A^{T}RC\\ \tau^{2}B^{T}RC \end{array} & \begin{array}{c} PD+\tau^{2}A^{T}RD\\ \tau^{2}B^{T}RD \end{array}\\ \begin{array}{cc} 0 & \;0 \end{array} & \tau^{2}C^{T}RC+Q & \tau^{2}C^{T}RD\\ \begin{array}{cc} 0 & \;0 \end{array} & 0 & \tau^{2}D^{T}RD-Q \end{array}]$$ (18)

when $\dot{V}<0$, by using Schur’s lemma, the above inequality is equivalent to LMI:


$$\begin{array}{c} \text{Y}<0\text{ when }\tau <\frac{1}{\left\|B\right\|+\left\|D\right\|}\sqrt{\frac{min\{\lambda \in R|det(Q-\lambda I)=0\}}{max\{\lambda \in \mathbb{R}|det(R-\lambda I)=0\}}} \end{array}$$ (19)

Then, it yields the stability condition:


$$J=[\begin{array}{cc} \frac{\partial a}{\partial v_{i}} & \frac{\partial a}{\partial {\Delta s}_{i}}\\ 1 & -T^{-1} \end{array}],tr(J)=\frac{\partial a}{\partial v_{i}}-T^{-1},\det(J)=-\frac{\partial a}{\partial {\Delta s}_{i}}-T^{-1}\frac{\partial a}{\partial v_{i}}$$ (20)

The eigenvalues λ of *J* should satisfy:


$$\lambda^{2}-tr(J)\lambda +\det(J)=0$$ (21)

For asymptotic stability, all eigenvalues must have negative real parts (tr(*J*)<0), det(*J*) should exceed zero, and ${tr}^{2}(J)-4\det(J)>0$. After Taylor expansion, neglecting higher-order small terms, the following equation can be derived.


$$-\tau {\left(\frac{f_{i}^{g}}{f_{i}^{v}}\right)}^{2}-{\left(\frac{f_{i}^{g}}{f_{i}^{v}}\right)}^{2}\frac{1}{f_{i}^{v}}+\frac{f_{i}^{g}f_{i}^{\Delta v}}{f_{i}^{v}f_{i}^{v}}+\frac{f_{i}^{g}}{f_{i}^{v}}(\sum_{j\in P_{i}}m_{j}\varpi_{i,j}j+\tau \frac{f_{i}^{g}}{f_{i}^{v}}\sum_{j\in P_{i}}m_{j}\varpi_{i,j}-\frac{1}{2})<0$$ (22)

This condition aligns with classical IDM stability bounds [35], confirming PIDM preserves stability under equilibrium.

## 4. Simulation analysis

This section aims to conduct simulation studies to illustrate the CAV platoon dynamics under different types of information flow topologies within V2V communication limitations. To better understand the complicated interactions between traffic dynamics and V2V communication information propagation, three sets of experiments based on information flow topologies are performed in this section.

**Fig 3:**
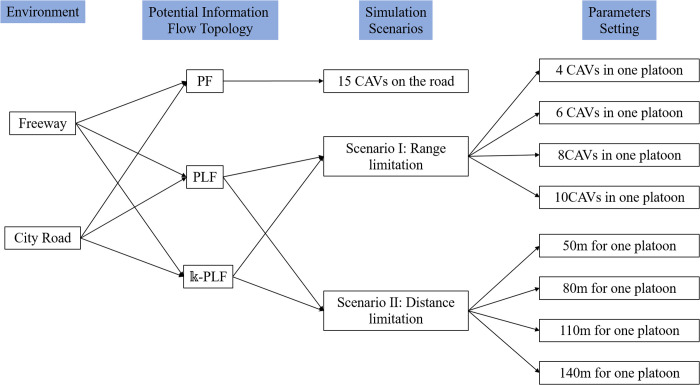
Overview of the numerical simulation.

Fig 3 summarizes a recapitulative structure for simulation scenarios and parameters setting. Scenario I can present the range limitation for CAVs, whereas scenario II demonstrates the distance limitation for CAVs under PLF and 𝕜-PLF topologies, respectively. Both scenarios include four cases and will be simulated in two environments: freeway and city road. Table 2 summarizes parameters adopted from the existing studies [18,35]. Unless otherwise specified, these parameters would not change.

### 4.1. CAVs with PF topology

Without loss of generality, we assume that in all scenarios, there are 15 CAVs at a speed of 24 m/s as the initial equilibrium speed traveling on the freeway and 10 m/s on the city road with a simulation cycle of 300 seconds. In addition, in the environment of the freeway, the uniform gap of them is 16.39m at the beginning of the simulation. During this time, a deceleration of 2 m/s^2^ spanning from the 10th second which is lasting 3 seconds, and an acceleration of 2 m/s^2^ spanning from the 130th second that lasts 3 seconds, are applied to the leading vehicle as small perturbations. Whereas, in the environment of city road, the uniform gap of them is 14.98m at the beginning of the simulation, with a deceleration of 1 m/s^2^ occurring at the 10th second and ending at the 13th second, and an acceleration of 1 m/s^2^ occurring at the 130th second and ending at the 133rd second. Then, until the end of the simulation, the velocity of the leading CAV is maintained.

**Fig 4:**
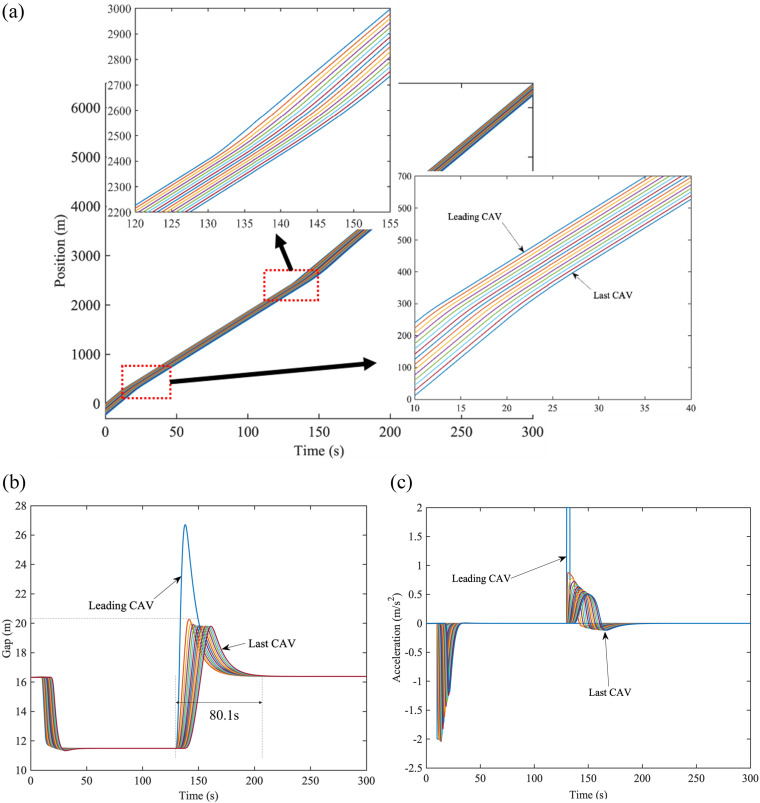
Position, gap, and acceleration profiles with PF topology on the freeway. (a) Position evolution, (b) Gap evolution, (c) Acceleration evolution.

**Fig 5:**
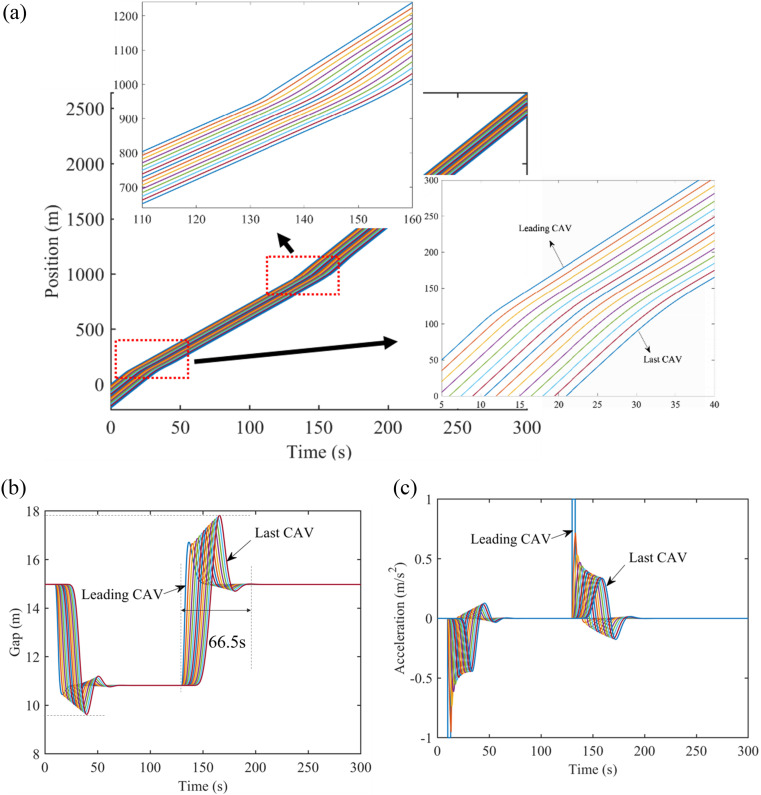
Position, gap, and acceleration profiles with PF topology on the city road. (a) Position evolution, (b) Gap evolution, (c) Acceleration evolution.

Fig 4 demonstrates the evolution of the position, velocity, gap, and acceleration of CAVs on the freeway under PF topology with reaction delay when they come across a small perturbation. Additionally, Fig 5 shows that of CAVs on the city road under PF topology. In terms of Fig 4(a) and Fig 5(a), CAVs can maintain safety during traveling. Since CAVs under PF topology can ignore the V2V communication range and distance constraints, the performance of CAVs with PF topology is set as the baseline. As shown in Figs 4(b) and 5(b), compared with the leading CAV decelerating, followers need to spend more time tracking the leading CAV when it suffers acceleration disturbance. Moreover, the time for all CAVs to return to the same gap is about 80.1 seconds on the freeway and 66.5 seconds on the city road. Note that, the effect of perturbation will be amplified as shown in Figs 4(c) and 5(c).

### 4.2. Scenario I: CAVs under V2V communication range limitation

In this scenario, we set four cases to illustrate the adverse effects of V2V communication range limitation on CAVs by designing four cases.

- **Case 1:** Only 4 CAVs are permitted to transform information in a platoon.
- **Case 2:** Only 6 CAVs are permitted to transform information in a platoon.
- **Case 3:** Only 8 CAVs are permitted to transform information in a platoon.
- **Case 4:** Only 10 CAVs are permitted to transform information in a platoon.

**Fig 6:**
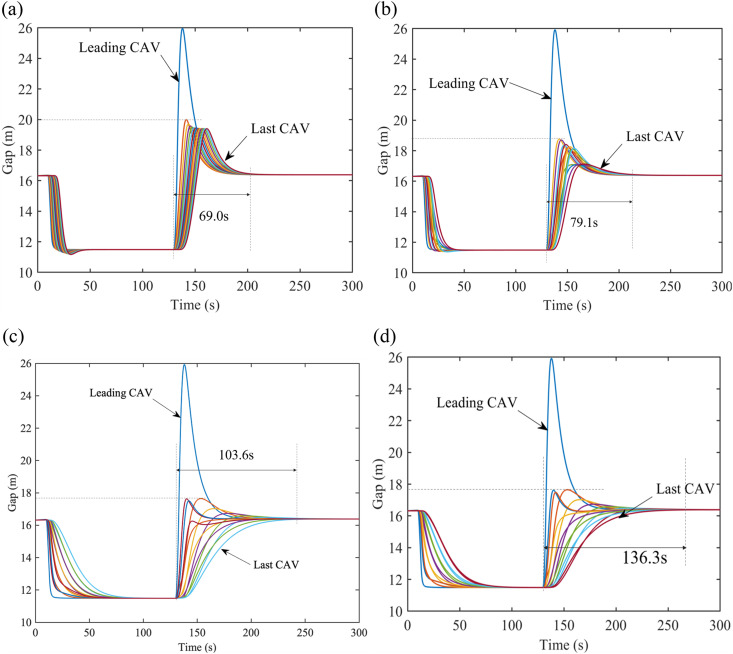
Gap profiles with PLF topology under V2V range limitation on the freeway. (a) Gap evolution with 4 CAVs in one platoon, (b) Gap evolution with 4 CAVs in one platoon, (c) Gap evolution with 8 CAVs in one platoon, (d) Gap evolution with 10 CAVs in one platoon.

Fig 6 presents changes in CAVs’ behaviors on the freeway under range limitation with PLF topology. It is apparent that the first two CAVs do not be influenced by the limitation, due to the fact each CAV only concerns with its preceding vehicles rather than its followers. Comparing Fig 6(a) with Fig 6(b)–(d), when communication range limitation is 4 CAVs, it takes the least time for CAVs to track the leading CAV. Additionally, this time is even less than CAVs with PF topology. Note that, it also takes more time for the following CAVs to track the leader with more CAVs are permitted in one platoon, but the speed and gap changes will not be amplified. This phenomenon results from that CAVs will be more sensitive than that with PF topology to perturbation and can react in advance when the size of a platoon is appropriate. But when there are more CAVs in a platoon, followers may obtain information of the leader far from them and do some unnecessary actions.

**Fig 7:**
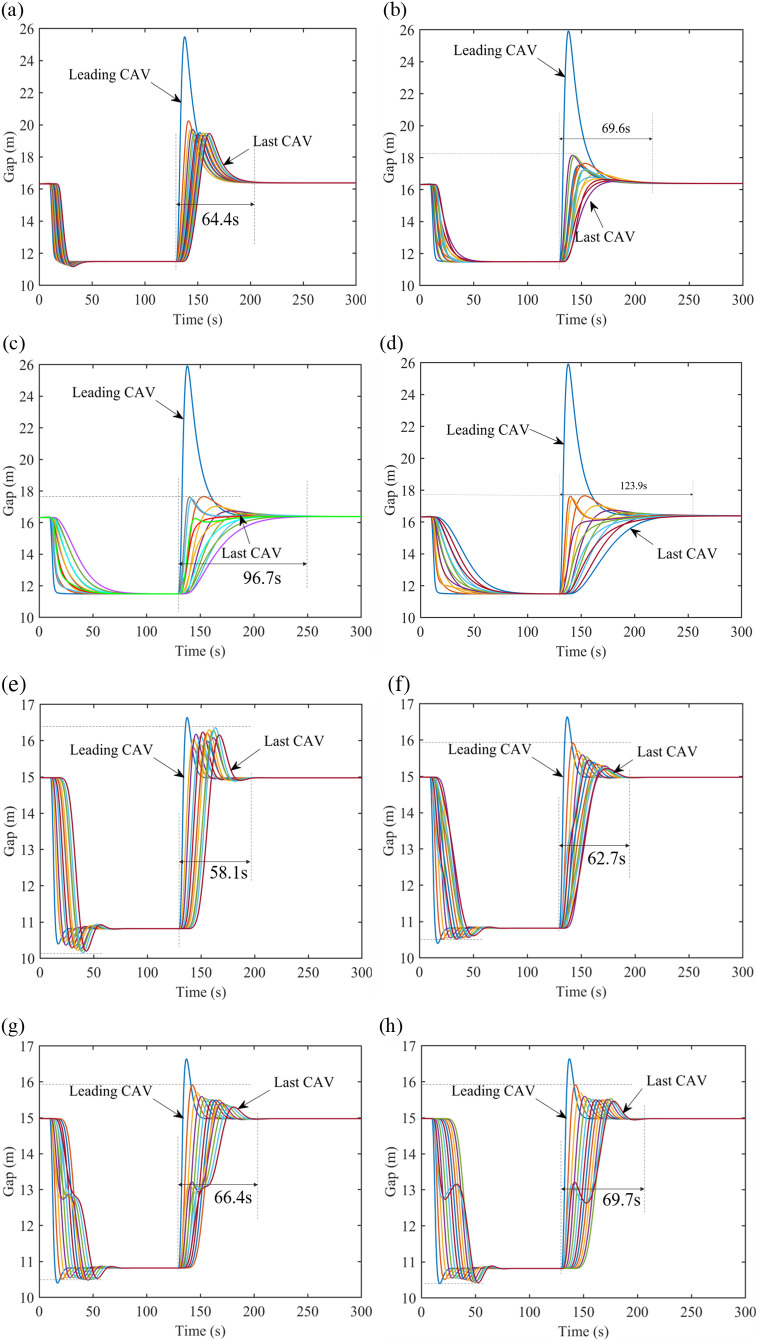
Gap evolution with 𝕜-PLF topology under V2V range limitation. (a) Gap evolution with 4 CAVs in one platoon on the freeway, (b) Gap evolution with 6 CAVs in one platoon on the freeway, (c) Gap evolution with 8 CAVs in one platoon on the freeway, (d) Gap evolution with 10 CAVs in one platoon on the freeway, (e) Gap evolution with 4 CAVs in one platoon on the city road, (f) Gap evolution with 6 CAVs in one platoon on the city road, (g) Gap evolution with 8 CAVs in one platoon on the city road, (h) Gap evolution with 10 CAVs in one platoon on the city road.

Fig 7 demonstrates the velocity profiles of CAVs under range limitation with 𝕜-PLF topology. The fluctuations of gaps in Fig 7(g) and (h) are caused by the PF topology between platoons and CAVs in the following platoons cannot react to the perturbances timely. Similar to the CAVs with PLF topology, more time will be spent but it can be smoother and steadier during driving, when the number of vehicles in one platoon is increasing. Nevertheless, 𝕜-PLF topology allows CAVs to obtain more information than PLF topology. Herein, in order to deal with the changes of gap and velocity, CAVs need less time to maintain the same spacing between each other, compared with CAVs with PLF topology in the same scenario in Fig 6.

**Fig 8:**
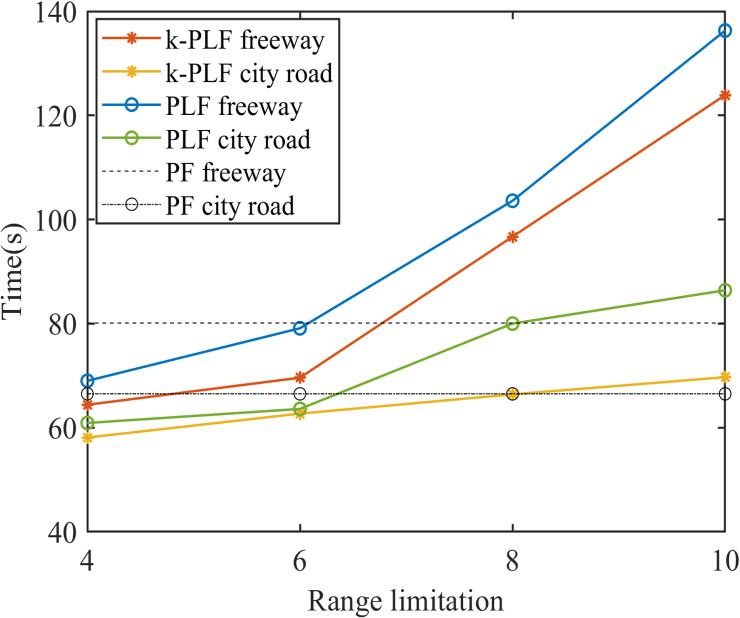
Time to restore to equilibrium under V2V range limitation.

Fig 8 shows the changes of the time to restore to equilibrium in different range limitations. As such, a conclusion can be drawn that with V2V communication range limitation, the management for CAVs is more flexible since less CAVs in each sub-platoon and CAVs can restore to equilibrium faster, if the range is small; in contrast, if the range is large, CAVs need more time to restore to equilibrium under perturbances. Further, with the range of V2V communication growing, CAVs will sacrifice some stability and efficiency for less states changes. The 𝕜-PLF topology’s performance superiority (Fig 8) is contingent upon idealized communication conditions. In scenarios with >20% packet loss, its multi-predecessor coordination may generate conflicting control inputs, as observed in [40,41]. Robustness to such failures will be explored in future work.

### 4.3. Scenario II: CAVs under V2V communication distance limitation

To better understand the impact of V2V communication, we divide V2V communication distance limitation into four cases in each environment depending on the vehicular dynamical model.

- **Case 1:** The most distance for the leader to send information in a platoon is 50 meters.
- **Case 2:** The most distance for the leader to send information in a platoon is 80 meters.
- **Case 3:** The most distance for the leader to send information in a platoon is 110 meters.
- **Case 4:** The most distance for the leader to send information in a platoon is 140 meters.

**Fig 9:**
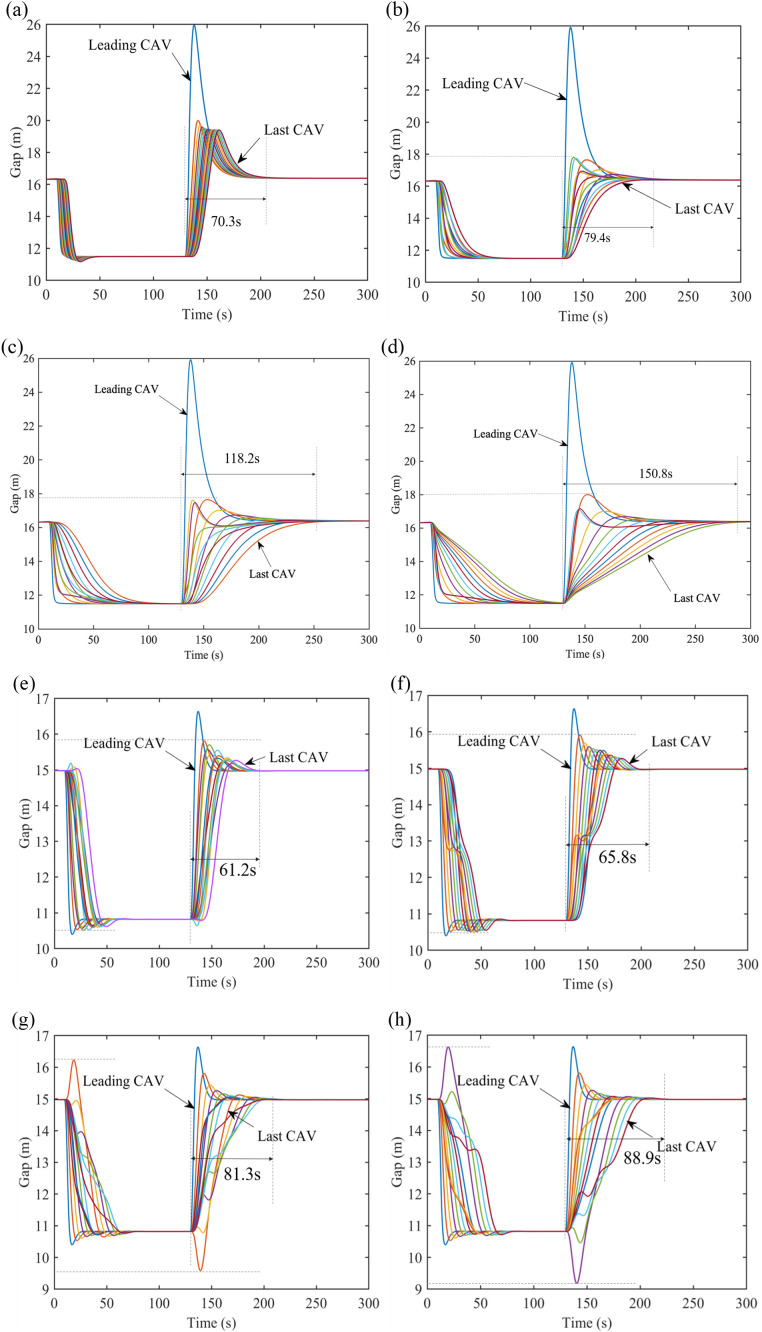
Gap evolution with PLF topology under V2V distance limitation. (a) Gap evolution with communication distance of 50m on the freeway, (b) Gap evolution with communication distance of 80m on the freeway, (c) Gap evolution with communication distance of 110m on the freeway, (d) Gap evolution with communication distance of 140m on the freeway, (e) Gap evolution with communication distance of 50m on the freeway on the city road, (f) Gap evolution with communication distance of 80m on the freeway on the city road, (g) Gap evolution with communication distance of 110m on the freeway on the city road, (h) Gap evolution with communication distance of 140m on the freeway on the city road.

**Fig 10:**
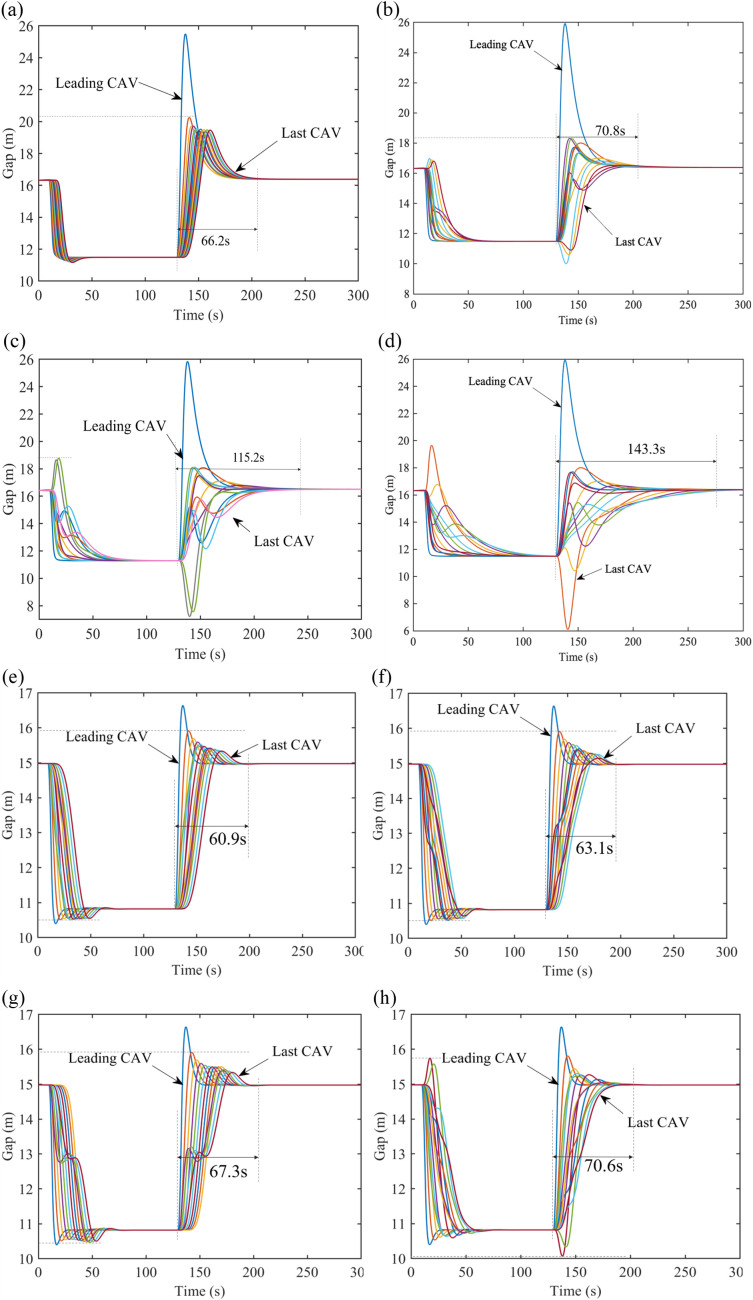
Gap evolution with 𝕜-PLF topology under V2V distance limitation. (a) Gap evolution with communication distance of 50m on the freeway, (b) Gap evolution with communication distance of 80m on the freeway, (c) Gap evolution with communication distance of 110m on the freeway, (d) Gap evolution with communication distance of 140m on the freeway, (e) Gap evolution with communication distance of 50m on the freeway on the city road, (f) Gap evolution with communication distance of 80m on the freeway on the city road, (g) Gap evolution with communication distance of 110m on the freeway on the city road, (h) Gap evolution with communication distance of 140m on the freeway on the city road.

In Figs 9 and 10, the gap changes of CAVs under distance limitation with PLF topology and with 𝕜-PLF topology are demonstrated. It is conspicuous that, with the length of distance limitation increasing, there might be fluctuations for the gaps between CAVs in the following platoons.

**Fig 11:**
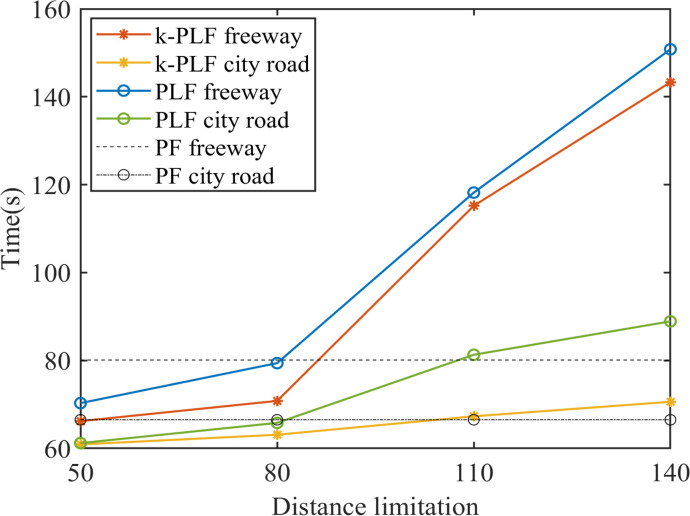
Time to restore to equilibrium under V2V distance limitation.

Additionally, CAVs are more likely to maintain stability on the city road than on the freeway where vehicles move faster. It is notable that the undulation is not amplified after being disturbed. The maximum gap and the maximum velocity in these cases are smaller than that under PF topology, as shown in Fig 11. Therefore, CAVs under distance limitation sacrifice a little time in exchange for smooth driving and can still restore to equilibrium timely.

On the whole, the communication distance for CAVs might change the number of vehicles in a platoon during driving, which will cause the behaviors (i.e., velocity, acceleration) of CAVs to produce unceasing fluctuation when the distance is increasing. Apart from that, CAVs with 𝕜-PLF topology can better maintain stability than CAVs with PLF topology and they both perform better than PF topology within a certain distance (in this paper is about 80m).

### 4.4. Scenario III: Heterogeneous traffic flow with CAVs and HDVs (Human-driving Vehicles) under V2V communication limitation

**Fig 12:**
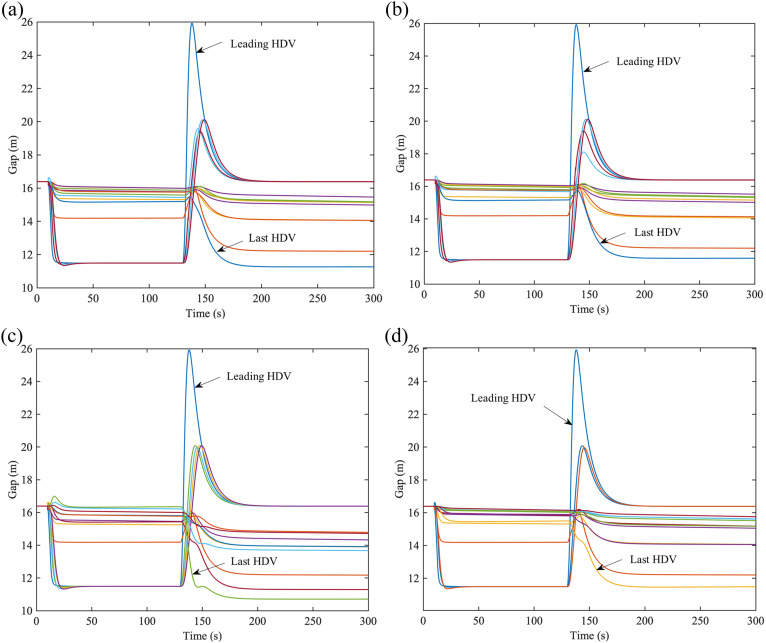
Gap evolution of heterogeneous platoon on the freeway. (a) Gap evolution with communication distance of 50m under PLF topology, (b) Gap evolution with communication distance of 50m under 𝕜-PLF topology, (c) Gap evolution with 4 vehicles in one platoon under PLF topology, (d) Gap evolution with 6 vehicles in one platoon under 𝕜-PLF topology.

To better understand the impact of V2V communication limitation, we introduce heterogeneous traffic flow with CAVs and HDVs scenario. Suppose there is a heterogeneous platoon of 15 vehicles on the freeway, where the first and the last vehicle is HDV and the rest are CAV. The result is shown in Fig 12.

Simulation results above demonstrate that CAVs can be affected by V2V communication limitations, and diverse types of limitations may result in diverse consequences. Therefore, these results also show that our proposed model can depict traffic behavior in the presence of V2V communication limitations. Further, it is more stable for CAV platoons on the city road than that on the freeway due to the velocity difference. Compared with PF topology, within certain limitation bounds, both PLF and 𝕜-PLF topologies can provide more stability with multi-predecessors sending information. Moreover, 𝕜-PLF topology works better than PLF topology under perturbances. However, with range limitation or distance limitation increasing, disturbance information will be sent to more vehicles and it takes more time for vehicles to restore to equilibrium with less amplitude of variation.

To rigorously evaluate platoon behavior under V2V communication limitations, we define three quantitative metrics:

**Recovery Time** ($T_{rec}$): Time elapsed from perturbation onset until speed fluctuations ($\sigma^{e}$) stabilize within 5% of the equilibrium value $v^{e}$.

**Peak Speed Fluctuation** ($\Delta v_{\max}$): Maximum absolute deviation from $v^{e}$ during the recovery phase.

**Stability Index** ($S_{idx}$): $S_{idx}=\frac{1}{T_{rec}}\int_{0}^{T_{rec}}\left|\frac{v(t)-v^{e}}{v^{e}}\right|dt\times 100\;\%$. Hence, lower $S_{idx}$ indicates faster convergence and smaller oscillations. Table 3 shows the performance comparison across topologies and scenarios via these quantitative metrics.

**Table 3:** Performance comparison across topologies and scenarios.

Scenario	V2V Limitation	Topology	$T_{rec}$ (s)	$\Delta v_{\max}$ (m/s)	$S_{idx}$ (%)
Freeway		PF	80.10	1.93	7.64
	4-vehicle limitation	PLF	76.25	1.81	7.17
		𝕜-PLF	74.89	1.44	5.70
	50m limitation	PLF	74.33	1.58	6.25
		𝕜-PLF	68.54	1.33	5.26
City road		PF	66.52	1.45	5.78
	4-vehicle limitation	PLF	62.47	0.75	4.13
		𝕜-PLF	61.93	0.59	3.61
	50m limitation	PLF	58.28	0.33	3.14
		𝕜-PLF	52.40	0.29	2.76
Heterogeneous traffic flow		PF	95.37	2.67	10.57
	4-vehicle limitation	PLF	92.24	2.03	8.04
		𝕜-PLF	91.46	1.78	7.05
	50m limitation	PLF	86.51	1.84	7.29
		𝕜-PLF	81.49	1.67	6.61

The proposed 𝕜-PLF topology consistently outperforms both PF and PLF across all scenarios, achieving the shortest recovery time ($T_{rec}$), lowest peak fluctuation ($\Delta v_{\max}$), and optimal stability index ($S_{idx}$). For instance, in heterogeneous traffic flow, 𝕜-PLF reduces $T_{rec}$ by ‌13.88%‌ (from 95.37s to 81.49s) and $S_{idx}$ by 37.5% (from 10.57% to 6.61%) compared to PF, demonstrating robust adaptability to mixed traffic interactions. Under range limitation, 𝕜-PLF achieves a 14.3% lower $\Delta v_{\max}$ than PLF in freeway scenarios (1.44 vs. 1.81 m/s), indicating enhanced oscillation suppression. The gap widens in city roads under distance limitation, where 𝕜-PLF attains $\Delta v_{\max}$=0.29 m/s (while PLF attains $\Delta v_{\max}$=0.33 m/s), suggesting distance-based constraints better align with urban dynamics. Performance degrades predictably with scenario complexity (Freeway < City road < Heterogeneous traffic), yet 𝕜-PLF maintains the smallest performance drop — e.g., $S_{idx}$ increases only from 2.76% (city road) to 6.61% (heterogeneous traffic), compared to PF’s 5.78% to 10.57%. These results strongly support 𝕜-PLF as a topology-adaptive solution for vehicular network stabilization, particularly under real-world constraints like mixed autonomy and dynamic disturbances.

## 5. Discussion

The mixed traffic experiments reveal a critical trade-off between topology complexity and environmental adaptability: while 𝕜-PLF leverages multi-predecessor data to optimize stability in homogeneous platoons, its reliance on consistent CAV feedback makes it vulnerable to HDV randomness. This aligns with findings in [36], where heterogeneous traffic necessitated decentralized control. PF’s single-predecessor dependency, though less efficient in ideal conditions, proves robust in low-CAV scenarios by minimizing error propagation. This suggests a ‘less is more’ principle for partial automation. Future systems could dynamically switch topologies based on real-time CAV penetration.

While this study advances our understanding of CAV platoon dynamics under V2V constraints, its scope is bounded by the following assumptions and technological dependencies: The analysis assumes single-lane platoons. Real-world scenarios involve multi-lane interactions (e.g., lane-changing, merging), which may amplify disturbance propagation [37,38]. Future work will integrate heterogeneous agent modeling frameworks [39] to address this gap. Moreover, a fixed reaction delay is assumed, ignoring stochastic packet loss and signal noise. Probabilistic delay models (e.g., Gamma-distributed delay time [40]) will be adopted in subsequent studies. Further, predefined PF/PLF/𝕜-PLF topologies lack adaptability to real-time traffic conditions. Dynamic topologies, such as those adjusting connectivity based on congestion levels [41], are critical for urban deployment but remain unexplored here. The study assumes mature V2V infrastructure with full coverage. Last but not the least, current deployments face challenges like cybersecurity risks [42] and fragmented connectivity. Hybrid architectures (V2V + LiDAR fusion) could mitigate these issues and warrant investigation.

## 6. Conclusion

To date, considerable research has demonstrated that CAV platoons hold great potential to maintain traffic stability and enhance safety stability through V2V communication. However, very few efforts have been undertaken to explore the impacts on CAV platoons under V2V communication limitations. In this study, we investigated the impacts of V2V communication limitations on the behaviors of CAV platoons with reaction delay under three different information flow topologies. Aim at capturing CAVs’ behaviors, this study develops platoon intelligent driver model integrating the dynamic communication topological structure and permitting information exchange among multiple CAVs, which differs from the classical IDM, denoted PIDM. In the end, numerical simulations are conducted to illustrate the evolution of vehicles’ behaviors under different V2V communication limitations.

The numerical experiments in this study have demonstrated that CAVs performance (i.e., time to restore to equilibrium) can be affected by both V2V communication range limitation and distance limitation. More specifically, small range limitation and small distance limitation would lead CAVs to restore to equilibrium faster under perturbances; in contrast, large range limitation and large distance limitation will cause the behaviors (i.e., velocity, acceleration) of CAVs to produce unceasing fluctuation and more restore time, since it might be dispensable for all CAVs in the sub-platoon (i.e., within communication range or distance) to response to perturbances. In addition, there are some findings during the experiment: firstly, CAVs with 𝕜-PLF topology can better maintain stability than CAVs with PLF topology and they both perform better than CAVs with PF topology within a certain range limitation or distance limitation but worse when limitation exceeds a certain threshold, since disturbance information will be sent to vehicles far from the vehicle subjective to disturbance and cause more time for vehicles to restore to equilibrium; secondly CAV platoons can be more stable with multi-predecessors sending information when the velocity of them is low (e.g., on the city road).

This study has several limitations that call for future research. One issue is the impact of V2V communication limitations on heterogeneous traffic. It is desirable to investigate the relationship between V2V communication limitation and CAV Market Penetration Rates. Another issue is that, multi-lanes are more common on roads, in reality. Future research is desired to explore the impacts of V2V communication limitation in scenarios with multi-lanes where overtaking and lane-changing maneuvers are allowed. Moreover, future research is also desired to employ real-world data to better depict the CAVs’ dynamics. This will assist our extrapolation to the general connected vehicle pool.

## Supporting information

S1 File Stability analysis. (PDF)
