# Supplementary material for: Modeling and analyzing V2V communication limitations impacts on connected and automated vehicle platoons
Source: PLoS One. 2025 Aug 18;20(8):e0328555. doi: 10.1371/journal.pone.0328555 (PMC12360585; doi:10.1371/journal.pone.0328555)
Supplement: S1 File — (PDF) [file pone.0328555.s001.pdf]

In the steady state, each follower in a platoon has the same distance gap and same speed as leader. Eq. (A 1) indicates the equilibrium result of Eq. (6) by $f_i(v_i^e, g_i^e, 0) = 0$.

$$\begin{cases} v_i(t) = v_i^e \\ \Delta s_i(t) = g_i^e \\ \Delta v_i(t) = 0 \\ a_i(t) = 0 \end{cases} \tag{A 1}$$

When the platoon is subjected to perturbation, then, let δv_i^e and δg_i^e denote the small deviation of the speed and space headway around the stationary situation:

$$\begin{cases} \delta v_i(t) = v_i^e - v_i(t) \\ \delta g_i(t) = \Delta s_i(t) - g_i^e \\ \Delta v_i(t) = 0 + \Delta(\delta v_i) \end{cases} \tag{A 2}$$

After substituting Eq. (A 2) into Eq. (6), and applying first-order Taylor expansion, the acceleration expression of CAV i under perturbation is as follows:

$$\begin{aligned} a_i(t) = \frac{\mathrm{d}v_i(t)}{\mathrm{d}t} &= f_i(v_i^e, g_i^e, 0) + f_i^v \delta v_i(t - \tau_i^v) \\ &+ f_n^g \sum_{j=1}^{P} k_j \left(\varpi_{i,j}(t-\tau)\right) \delta g_{i-j+1}(t-\tau) \\ &+ f_n^{\Delta v} \sum_{j=1}^{P} k_j \left(\varpi_{i,j}(t-\tau)\right) \delta v_{i-j+1}(t-\tau) \end{aligned} \tag{A 3}$$

where

$$f_i^v = \left.\frac{\partial f_i}{\partial v_i}\right|_{(v_i^e, g_i^e)}, f_i^g = \left.\frac{\partial f_i}{\partial s_i}\right|_{(v_i^e, g_i^e)}, f_i^{\Delta v} = \left.\frac{\partial f_i}{\partial \Delta v_i}\right|_{(v_i^e, g_i^e)} \tag{A 4}$$

where f_i^v, f_i^s, and $f_i^{\Delta v}$ represent the partial differential equations for speed, gap, speed difference at the equilibrium arguments Eq. (A 1), respectively. Based on the principle of linear system superposition, let:

$$\begin{cases}\delta v_i(t) = V_i e^{i(\omega i)+\lambda t} \\ \delta g_i(t) = G_i e^{i(\omega i)+\lambda t}\end{cases} \tag{A 5}$$

where $i(\cdot)$ stands for the imaginary part, V_i and G_i are constant which are equal to V and G. Additionally, $\mathrm{G} = \frac{\mathrm{V}(e^{-i(\omega)}-1)}{\lambda}$ can result in:

$$\begin{pmatrix} \lambda & (1-e^{-i(\omega)}) \\ -f_i^g \sum_{j=1}^{P} k_j\left(\varpi_{i,j}(t-\tau)\right) e^{-\lambda\tau - i(\omega(j-1))} & \left[\lambda - (f_i^v e^{-\lambda\tau} - f_i^{\Delta v} \sum_{j=1}^{P} k_j\left(\varpi_{i,j}(t-\tau)\right) e^{-i(\omega j)-\lambda\tau}(1-e^{i(\omega)})\right] \end{pmatrix} \begin{pmatrix} \mathrm{G} \\ \mathrm{V} \end{pmatrix} = 0 \tag{A 6}$$

The system is linear stable only when the determinant of the coefficients matrix of Eq. (A 6) is equal to zero. Hence, a quadratic characteristic equation is needed to be satisfied:

$$\begin{aligned} &\lambda^2 - \left[(f_i^v e^{-\lambda\tau} - f_i^{\Delta v} \sum_{j=1}^{P} k_j\left(\varpi_{i,j}(t-\tau)\right) e^{-i(\omega j)-\lambda\tau}(1-e^{i(\omega)})\right]\lambda \\ &+ \left[(1-e^{-i(\omega)}) f_i^g \sum_{j=1}^{P} k_j\left(\varpi_{i,j}(t-\tau)\right) e^{-\lambda\tau - i(\omega(j-1))}\right] = 0 \end{aligned} \tag{A 7}$$

Then, introduce the power series solution of m:

$$m = i(\omega)m_1 + i(\omega^2)m_2 + \cdots \tag{A 8}$$

where m_1 and m_2 are real coefficients. By solving the above algebraic formula, m_1 and m_2 can be calculated as:

$$m_1 = f_i^g / f_i^v \tag{A 9}$$

$$m_2 = -\tau\left(\frac{f_i^g}{f_i^v}\right)^2 - \left(\frac{f_i^g}{f_i^v}\right)^2 \frac{1}{f_i^v} + \frac{f_i^g f_i^{\Delta v}}{f_i^v f_i^v} + \frac{f_i^g}{f_i^v}\left(\sum_{j=1}^{P} k_j\,\varpi_{i,j} j + \tau\frac{f_i^g}{f_i^v}\sum_{j=1}^{P} k_j\,\varpi_{i,j} - \frac{1}{2}\right) \tag{A 10}$$

According to previous studies [35], the partial differentials:

$$f_i^v > 0, f_i^g > 0, \text{ and } f_i^{\Delta v} < 0 \tag{A 11}$$

the linear stability for the traffic flow is obtained:

$$-\tau\left(\frac{f_i^g}{f_i^v}\right)^2 - \left(\frac{f_i^g}{f_i^v}\right)^2 \frac{1}{f_i^v} + \frac{f_i^g f_i^{\Delta v}}{f_i^v f_i^v} + \frac{f_i^g}{f_i^v}\left(\sum_{j=1}^{P} k_j\, \varpi_{i,j} j + \tau \frac{f_i^g}{f_i^v}\sum_{j=1}^{P} k_j\, \varpi_{i,j} - \frac{1}{2}\right) < 0 \tag{A 12}$$

As such, Eq. (A 12) can be degraded into the stability situation for CAV platoon without reaction delay. In this study, IDM is leveraged to capture the performance of CAV platoon. The partial differentials of IDM are representing as:

$$\begin{cases} f_i^v = -\dfrac{\delta\alpha (v_i^e)^{\delta-1}}{{v_f}^{\delta}} - \dfrac{2\alpha T\left[1-\left(v_i^e/v_f\right)^{\delta}\right]}{s_0 + T v_i^e} \\ f_i^g = 2\alpha\dfrac{\sqrt{1-\left(v_i^e/v_f\right)^{\delta}}\left[1-\left(v_i^e/v_f\right)^{\delta}\right]}{s_0 + T v_i^e} \\ f_i^{\Delta v} = -\sqrt{\dfrac{\alpha}{\beta}}\dfrac{v_i^e\left[1-\left(v_i^e/v_f\right)^{\delta}\right]}{s_0 + T v_i^e} \end{cases} \tag{A 13}$$

where $f_i^v < 0$ ensures negative feedback that vehicles reduce acceleration when exceeding equilibrium speed v_i^e, and can promotes local stability; $f_i^g > 0$ enforces positive feedback on spacing which restoring equilibrium; $f_i^{\Delta v} < 0$ implies vehicles decelerate when approaching a slower predecessor, preventing collisions. These partial derivatives define the sensitivity of the vehicle's acceleration to state variations around equilibrium:

- $f_i^v < 0$: Negative feedback on speed error. An increase in v_i reduces acceleration, promoting local stability by counteracting speed deviations.
- $f_i^g > 0$: Positive feedback on spacing error. A decrease in gap Δs_i triggers deceleration,

enforcing equilibrium spacing.

- $f_i^{\Delta v} < 0$: Damping effect on relative speed. A positive Δv_i (approaching predecessor) induces deceleration, suppressing oscillations.

Collectively, these terms govern the restoring forces that counteract perturbations, where $|f_i^v|$ and $|f_i^g|$ determine convergence strength, and $f_i^{\Delta v}$ controls disturbance damping.

Substituting the PIDM parameters from Table 2 (v_i^e=24m/s, δ=4, α=1m/s^2, β=2m/s^2, v_f=120km/h), into Eq. (A13) yields: f_i^v≈-0.38, f_i^g≈0.12, $f_i^{\Delta v}$≈−0.21. For PF topology, the delay threshold is τ_{max}≈0.85s via Eq. (A12). Under 𝕜-PLF with 10 vehicles, τ_{max} drops to 0.52s. This aligns with our simulation setting τ=0.2s<τ_{max}, explaining why all topologies maintained stability in Section 4.
